# DNA Replication: From Radioisotopes to Click Chemistry

**DOI:** 10.3390/molecules23113007

**Published:** 2018-11-17

**Authors:** Anna Ligasová, Karel Koberna

**Affiliations:** Faculty of Medicine and Dentistry, Institute of Molecular and Translational Medicine, Palacký University in Olomouc, Hněvotínská 5, 779 00 Olomouc, Czech Republic

**Keywords:** click chemistry, nucleoside and nucleotide analogues, indirect immunocytochemistry, isotopes

## Abstract

The replication of nuclear and mitochondrial DNA are basic processes assuring the doubling of the genetic information of eukaryotic cells. In research of the basic principles of DNA replication, and also in the studies focused on the cell cycle, an important role is played by artificially-prepared nucleoside and nucleotide analogues that serve as markers of newly synthesized DNA. These analogues are incorporated into the DNA during DNA replication, and are subsequently visualized. Several methods are used for their detection, including the highly popular click chemistry. This review aims to provide the readers with basic information about the various possibilities of the detection of replication activity using nucleoside and nucleotide analogues, and to show the strengths and weaknesses of those different detection systems, including click chemistry for microscopic studies.

## 1. Introduction

The DNA molecule was discovered in 1869 by the Swiss chemist Friedrich Miescher [1]. However, it was not until 1944 that it was first hypothesized that DNA molecules are the carriers of genetic information [2]. The concept that DNA serves as a carrier of genetic information was proved by Hershey and Chase in 1952 [3], and already in 1953, based on the X-ray analyses, the structure of the DNA molecule was described [4]. These findings resulted in a subsequent huge upsurge of interest in DNA, and in an intense increase of knowledge about the processes connected with its organization, repair, replication, and expression of genetic information. Through the following years, it was, for example, discovered that eukaryotic chromosomal DNA is replicated via a large number of DNA segments known as *replicons*, which are continuously activated in the S phase, and that their replication is ensured by couples of “sister” replisomes. It was also described that replication proceeds from origins of replication bidirectionally through the agency of two replication forks, and terminates when the forks of neighboring replicons converge (reviewed e.g., in [5,6,7,8]).

Inseparably, the methodological approaches used during the description of all these processes were continually being developed and improved. Many of these approaches also found use in medical research and in the diagnostics of various diseases. In addition, the DNA became the target of the treatment of patients suffering from various forms of malignant tumors. 

One of the important methodology areas that has remained a remarkable development over the years is the field dealing with the detection of replicational activity. Over several decades, the initial methods of the detection of replicational activity were improved, modified, or substituted by others that were shorter, more sensitive, or less toxic. 

In this review, we will summarize the information on the possibilities of the detection of replication activity using various nucleoside and nucleotide analogues, and show the strengths and weaknesses of the different detection systems, including click chemistry for microscopic studies.

## 2. Labeling of Replicated DNA by Isotopically Labeled Nucleosides

The majority of methods used for the detection of replication activity are based on the incorporation of modified nucleosides into replicated DNA and their subsequent detection. The first marker nucleosides used radioisotopes for their labeling and detection. An example of such isotopically-labeled nucleosides are N^15^-thymidine [9] and C^14^-thymidine [10,11,12]. This technology showed that, e.g., thymidine is a precursor for DNA, but not for RNA [9].

In 1957, Taylor and colleagues developed a method where they used H^3^-thymidine for the labeling of replicated DNA in the bean root, and autoradiography for its detection. The approach used revealed differences in the labeling of particular chromosomal chromatids after the second replication cycle, where only one of each two chromatids were labeled; this contributed significantly to the discovery of the semi-conservative replication of DNA [13]. The autoradiography is based on the formation of silver grains in films. The radiation from radioisotopes interacts with the photographic emulsion in contact with the sample, thus providing an image of the distribution of the radioactivity in the sample. The word “autoradiography” comes from the fact that the sample is not placed between the source of radiation and the detector, as it is in radiography, but itself contains the source of radiation [14].

Autoradiography enabled, in the following years, the description of many other processes connected with DNA synthesis. Using autoradiography, John Cairns showed that the replication of bacterial DNA proceeded by replication fork, and that during DNA replication unwinding of DNA occurred [15]. He obtained similar results several years later in human HeLa cells [16]. In the same year, Huberman and Riggs used autoradiography for the detection of DNA replication on stretched DNA fibers in mammalian cells [17], and Ribas-Mundó for the description of three patterns of DNA replication in normal human leukocyte cells [18]. Only a little later, the radioactive labeling enabled the revelation that the lagging strand of DNA is replicated by short Okazaki fragments [19,20,21,22].

Labeling of replicated DNA using H^3^-thymidine followed by autoradiography was used not only for light microscopy detections, but also for electron microscopy studies. In this respect, it was, for example, shown that euchromatin is replicated early in the S phase, whereas heterochromatin replicates later in the S phase; as such, the sites of DNA synthesis were localized [23,24].

Although autoradiography enabled the discovery of many important features related to DNA replication, this technique was gradually forced out by new approaches. Probably the most important limitation of the autoradiography approach is the fact that it is time consuming and exhibits low resolution, caused by the distribution of the signal over a relatively large area. In this respect, the exposition time necessary to obtain a significant signal can be up to several months. Another significant drawback is the relatively high signal/background ratio. Finally, the autoradiography approach requires manipulation with radioactive material, and therefore, a whole range of safety regulations have to be observed. The mentioned reasons led to the development of alternative systems. The first such broadly-used system was based on the halogenated analogs of nucleosides.

## 3. Halogenated Analogs of Nucleosides as a Tool for the Research of DNA Synthesis

The effectiveness of the phosphorylation, and incorporation of non-isotopically labeled nucleosides into DNA, is usually lower than in the case of isotopically-labeled nucleosides. Therefore, it is not surprising that only a small part of the tested, structurally changed nucleosides can serve as a reliable marker of replicated DNA.

In this respect, the halogen derivatives of thymidine represent the first widely-used alternative to the isotopically-labeled nucleosides. These thymidine analogs are modified in the 5 position of the thymine ring by halogen atoms (bromine, fluorine, chlorine and iodine). Although the most widely-known representative of this group: 5-bromo-2′-deoxyuridine (BrdU) was synthesized already in 1955 by Beltz and Visser [25], more than 15 years was necessary to develop an efficient way to use it in non-isotopic detection in cellular DNA.

BrdU is very efficiently incorporated into DNA, and its toxicity is relatively low. In this respect, it was shown that BrdU impact on the growth of bacteria *E. coli* K-12 is approximately thirty times lower than the impact of 5-hydroxy-2′-deoxyuridine [25]. The incorporation of BrdU into bacterial DNA was demonstrated for the first time in 1960 [26], and into mammalian DNA in 1959 [27,28,29].

Until the discovery of specific antibodies, the detection systems of BrdU in DNA were mainly based on autoradiography [27,30]. In 1971, specific antibodies raised against BrdU were produced [31], and the immunochemical detection of the incorporated BrdU was described in denaturated DNA [31]. Four years later, the method of the immunofluorescence in in situ detection of incorporated BrdU in nuclear DNA was developed [32]. Besides the immuno-detection of BrdU, the detection system based on the finding that the fluorescence of Hoechst stains bound to DNA is suppressed by incorporated BrdU was used [33]. Although this system does not require any special DNA treatment, it is not suitable for common microscopic studies. On the other hand, it was successfully used for the analysis of the cell cycle using flow cytometry [34,35,36].

As BrdU and also other halogen derivatives of thymidine are inaccessible in double-stranded DNA for reactions with antibodies, it is necessary to use special treatments to make them accessible for such reactions. In this respect, the protocols based on the use of acid or hydroxide treatments are very common approaches for BrdU detection [37,38,39,40,41]. While the hydrochloric-acid solutions results in depurination and cleavage of the DNA, the treatment with the sodium hydroxide results in a loosening of the DNA structure as a consequence of the deprotonation of the nucleobases [42]. The use of strong acids also leads to the partial destruction of cellular components. For example, the use of highly-concentrated HCl (usually 1.5 M–4 M) leads to the destruction of many nuclear antigens [38,40,42,43]. In the case of the concurrent labeling of cellular DNA by DNA dyes such as DAPI, Hoechst dyes, or propidium iodide, such treatment can substantially decrease their signal [42,44]. Protocols based on the use of strong alkalis, although commonly used in lower concentrations, exhibit a similar negative impact on the cellular structures [40,42].

**Figure 1:**
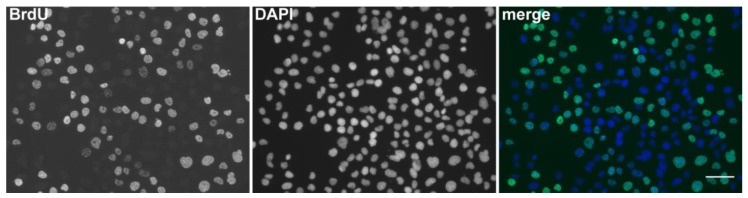
The detection of BrdU using DNase I and exonuclease III. Examples of microscopic images of cell nuclei labeled for 5 min with BrdU and fixed with formaldehyde. The DNA was labeled by DAPI. The scale bar—50 μm. Adapted with permission from Ligasová et al., 2017, Looking for ugly ducklings: The role of the stability of the BrdU-antibody complex and the improved method of the detection of DNA replication. PLoS ONE 12(3): e0174893 [47].

The above-mentioned problems led to the development of alternative approaches of BrdU detection. One common alternative method is based on the cleavage of DNA by nucleases. Either a type of nuclease, or a mixture of various nucleases, is used to generate short single-stranded tracts accessible for binding the anti-BrdU antibodies [38,40,45,46]. The use of nucleases is one of the approaches that does not exhibit a strong negative effect on the cellular structure. The approach based on nucleases was further optimized from the viewpoint of its effectivity, and currently it is one of the best methods for the detection of DNA replication, even if a short labeling pulse of BrdU is used (Figure 1), enabling co-localization studies including those with fluorescent proteins as well [47].

A simple and very efficient method of BrdU detection providing high signal/background ratio is based on the use of low concentration of HCl and exonuclease III [48]. In this case, the breaks in the DNA are introduced by the low concentration of hydrochloric acid followed by the subsequent enzymatic extension of these breaks using exonuclease III. This approach enables, at least to some extent, co-localization studies with fluorescent proteins. Concerning the cellular proteins, the immunocytochemical detection of proteins can vary from protein to protein and from antibody to antibody; therefore, the impact of the method on the localization and signal intensity of particular protein should be tested first [48].

Another alternative for the detection of BrdU is the cleavage of DNA using monovalent copper ions. This method is based on the oxidative attack at the deoxyribose moiety by monovalent copper in the presence of oxygen [42]. This approach provides a high signal/background ratio, is quickly feasible, does not require any special equipment, and preserves at least some type of protein antigens [42].

A further method of BrdU detection is based on the photolysis of BrdU-labeled DNA followed by the detection of the induced breaks [49]. This method uses illumination of cells with UV light to selectively photolyze DNA with the incorporated BrdU [36,49,50]. Heat denaturation is another way to make incorporated BrdU accessible for antibodies. In this case, cellular DNA can be denatured at 80 °C–95 °C, before incubation with anti-BrdU antibody [36,40].

**Figure 2:**
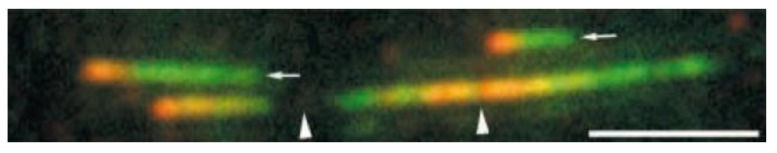
Labeling patterns of newly replicated DNA visualized on DNA fibers. DNA was first labeled with CldU for 10 min (green) and then with IdU for 5 min (red). Two isolated elongating forks (arrow) and replicons with initiation and termination sites (arrowheads) are shown. Bar = 5 µm. Adapted with permission from Malínský et al., 2001, The supply of exogenous deoxyribonucleotides accelerates the speed of the replication fork in the early S phase. Journal of Cell Sciences 114, 747–750 [54].

Besides BrdU, nucleosides modified with chlorine (5-chloro-2′-deoxyuridine, CldU) [51] or iodine (5-iodo-2′-deoxyuridine, IdU) [52] can be used. However, the use of both latter mentioned nucleoside analogs is usually restricted to some special cases. IdU and CldU were and still are used mainly for the double labeling of both DNA in cells [53], and also on DNA fibers (Figure 2) [54]. The latter mentioned method is presently called *DNA combing* or *DNA fiber analysis*, and is used to study the DNA replication dynamics [55,56]. In the case of these two nucleoside analogs, it is also necessary to treat the DNA before the reaction with the antibodies.

The double labeling of replicating DNA is based on the use of two different clones of primary antibodies raised against BrdU. One of these antibodies reacts also with CldU but not with IdU. The second antibody clone reacts with both CldU and IdU. However, after washing in a buffer with a high salt concentration, the antibodies linked to CldU-labeled DNA are removed [53].

**Figure 3:**
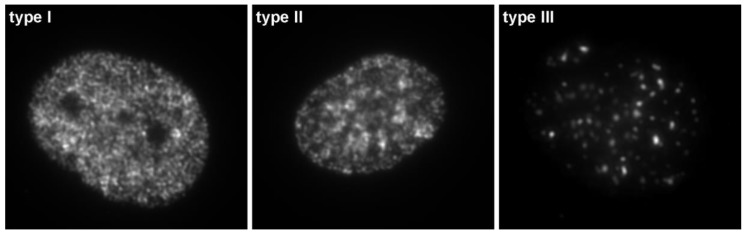
Three replication patterns visualized by BrdU immuno-detection during the S phase. The HeLa cells were labeled with BrdU for 10 min, fixed (2% formaldehyde, 10 min), permeabilized (0.2% Triton X-100, 10 min), and treated with 4N HCl (20 min). The incorporated BrdU was visualized using anti-BrdU antibody and Cy3 labeled anti-mouse antibody. **Type I**—large number of small replication sites characteristic for early S phase; **type II**—replication sites concentrated at the periphery of the nucleus and nucleoli (mid S phase); **type III**—large replication sites over heterochromatin (late S phase).

The approaches based on the immuno-detection of halogen derivatives are very important procedures used for the detection of the replicational activity of cells, and serve as an important tool for the description of cellular proliferation and cell cycle by microscopy or flow cytometry as well, e.g., [57,58,59,60,61,62,63]. They also enabled researchers to collect a new data about the organization of replication. In this respect, it was, for example, proven that during the S phase, three basic replication patterns can be observed (Figure 3), or if described in more detail, five patterns can be observed in mammalian cells [64,65,66].

BrdU labeling also allowed the determination of sites of DNA synthesis on chromosomes with relation to chromosome bands. In this respect, R-bands correspond to the early-replicated chromatin, and G-bands to the late-replicated chromatin [67].

Using the double labeling by IdU and CldU, it was, for example, shown that the addition of exogenous deoxyribonucleotide triphosphates increases the speed of DNA replication only at the beginning of the S phase. Later in the S phase, this addition has no effect on the speed of replication [54]. The double labeling of cells with IdU and CldU was also applied to the analysis of the cell proliferation, temporal analysis of DNA replication, or cell cycle kinetic [53,68,69].

**Figure 4:**
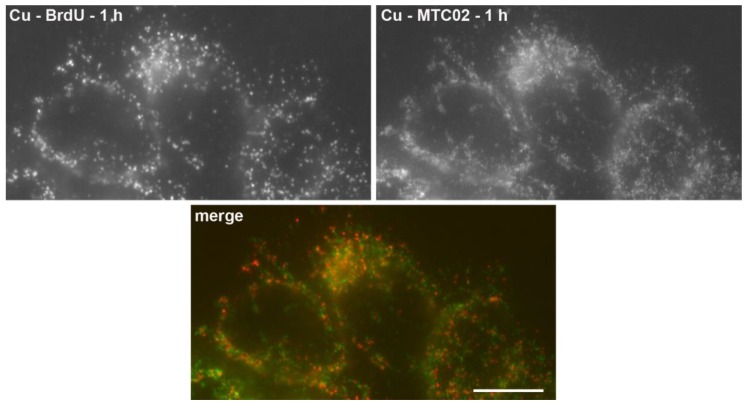
The detection of the BrdU-labeled DNA in mitochondria. The detection of the BrdU-labeled DNA (red in the color image) after a 1-hour BrdU pulse in cells treated with 4 mM copper(I) for 60 s followed by exonuclease III cleavage is shown. A simultaneous co-localization with the mitochondrial marker MTC02 has been performed (green in the color image). Bar: 10 µm. Adapted with permission from Ligasová et al., 2012, Atomic Scissors: a new method of tracking the 5-bromo-2′-deoxyuridine-labeled DNA in situ. PLoS ONE 7(12): e52584 [42].

BrdU labeling is used not only for the description of the sites of nuclear DNA synthesis, but also for the analysis of sites with replicated mitochondrial DNA (Figure 4). In this case as well, the samples have to be treated before BrdU detection. The most common treatment is based on the use of HCl [70,71,72], although the cleavage of DNA by monovalent copper ions can provide higher efficiency [42]. If necessary, the BrdU signal can be further amplified [71].

Most studies with marker nucleosides are performed using light microscopy; however, these nucleosides can also be employed in ultrastructural studies using electron microscopy. In this respect, BrdU was used for example for the ultrastructural description of replication sites [41] or replication patterns [73,74], or the association of endogenous BCL6 with replication foci [75].

## 4. Click Chemistry and Its Use in DNA Replication Research

### 4.1. 5-Ethynyl-2′-deoxyuridine

**Figure 5:**
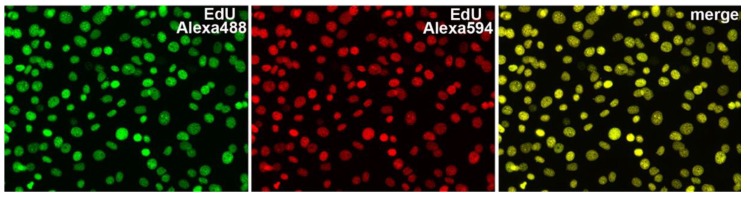
Labeling of DNA using EdU. NIH 3T3 cells labeled by incubation overnight with 2 μM of EdU were fixed and reacted successively with 10 μM of Alexa488-azide and 10 μM of Alexa594-azide, respectively. The cells were imaged by fluorescence microscopy. (**Left**) Alexa488-azide stain. (**Center**) Alexa594-azide stain. (**Right**) Overlay of the Alexa488 and Alexa594 images. Adapted with permission from Salic, A., Mitchison, T.J., 2008, A chemical method for fast and sensitive detection of DNA synthesis in vivo. Proc Natl Acad Sci U S A 105, 2415–2420, Copyright (2008) National Academy of Sciences U.S.A [76].

Because of the necessity to disrupt the structure of double-stranded DNA in the case of halogen derivatives of thymidine, other possibilities of detection of replicating DNA were investigated. In 2008, Salic and Mitchinson published an alternative method for the labeling of DNA replication. They used 5-ethynyl-2′-deoxyuridine (EdU) and click reaction with Alexa-azide stains for the detection of replicating DNA (Figure 5) [76].

**Figure 6:**
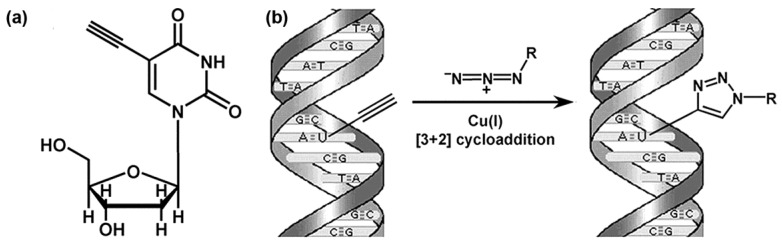
The use of EdU to label DNA in cells. (**a**) Structure of EdU. (**b**) Scheme of the click reaction for detecting EdU incorporated into cellular DNA. The terminal alkyne group, exposed in the major groove of the DNA helix, readily reacts with an organic azide (R can be any fluorophore, hapten, electron-dense particle, quantum dot, etc.) in the presence of catalytic amounts of Cu(I). Adapted with permission from Salic, A., Mitchison, T.J., 2008, A chemical method for fast and sensitive detection of DNA synthesis in vivo. Proc Natl Acad Sci U S A 105, 2415–2420, Copyright (2008) National Academy of Sciences U.S.A. [76].

This modified nucleoside has a terminal alkyne group in the 5 position, instead of a methyl group (Figure 6a). The EdU was synthesized already at the end of the 1970s [77,78]. Initially, its impact on viruses and cancer cells was analyzed. It was found that EdU has an anti-HSV-1 and HSV-2 (Herpes simplex virus) effect and also an impact against the vaccinia virus or cytomegalovirus. Concurrently, the effective concentration proves to be too toxic for non-infectious cells [79,80,81,82]. In 2007, EdU was successfully tested as a possible inhibitor of the cell growth of human breast cancer cells [83]. Surprisingly, for a long time, EdU was believed to not be incorporated into DNA [84]. The main mechanism of its toxicity was attributed to its inhibition of the enzyme thymidylate synthase [84,85]. Not until 2008 did Salic and Mitchinson show that EdU is strongly incorporated into the DNA of mammalian cells [76].

The detection of EdU incorporated into DNA is performed through a Cu(I)-catalyzed [3 + 2] cycloaddition (click chemistry, Figure 6b) when the terminal alkyne group reacts with azides, e.g., fluorescently labeled [76,86,87]. Thanks to the simplicity, quickness, and mainly due to the fact that EdU detection does not require any DNA treatment, EdU quickly became a widely-used alternative for the detection of the replicational activity of cells. EdU is presently broadly used. Examples include studies focused on the analysis of DNA replication, S-phase progression, the monitoring of cells in subsequent cell cycles, cellular proliferation, and other processes connected with DNA synthesis e.g., [88,89,90,91].

The massive usage of EdU in various studies concerning DNA synthesis, cellular proliferation, and related processes confirmed the previously-known fact that EdU is highly toxic in the commonly-used concentrations [92,93,94,95]. Some studies showed that EdU incorporation can lead to DNA breaks followed by cell death [92,94,95]. Other data strongly indicated that EdU toxicity correlates with the efficiency of EdU incorporation into DNA [93]. This toxicity was accompanied by the deformation of the cell cycle, the slowdown of the S phase, and the induction of interstrand crosslinks [93]. Taken together, all these data showed that EdU is extremely useful for short term experiments, but it is not suitable for long-term studies [92,93].

EdU and its detection using a click reaction with copper ions is not compatible with live cell imaging [96,97]. Copper ions are highly toxic for cells [76,96]. It was also shown that monovalent copper ions in the presence of oxygen causes the significant cleavage of DNA [42]. Moreover, the click reaction results in the destruction of the fluorescence of the proteins such as GFP or R-phycoerythrin [97]. Therefore, special protocols preventing the degradation of fluorescent proteins have been developed [98,99].

**Figure 7:**
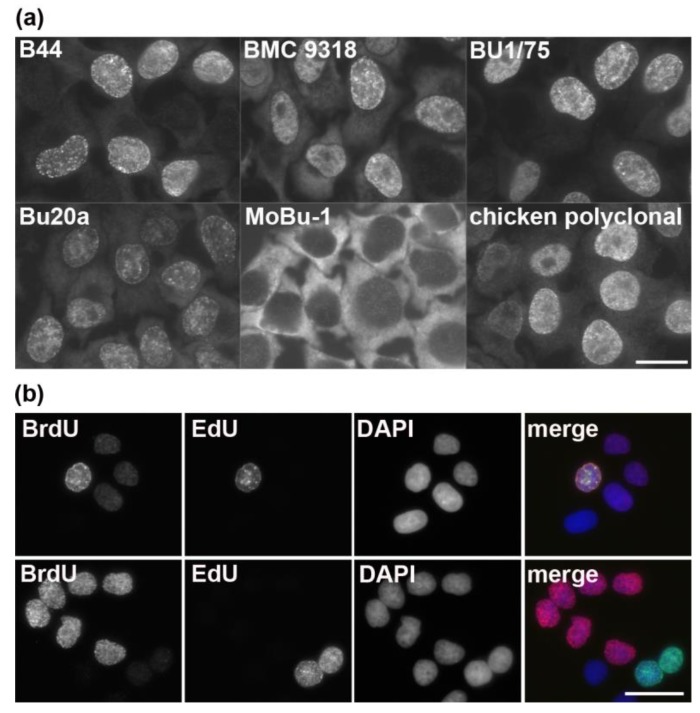
The detection of EdU in fixed HeLa cells and the simultaneous localization of BrdU and EdU. (**a**) The picture shows examples of the detection of EdU in fixed and permeabilized cells with six anti-BrdU antibodies after a ten-minute EdU labeling pulse. Note that only the clone MoBu-1 exhibits no signal. Bar: 20 µm. (**b**) The results of simultaneous localization of BrdU and EdU of cells labeled for 5 min with BrdU and then after 13 h for 20 min with EdU is shown. The upper set of images represents the detection of both signals without the application of the blocking step by means of 2 mM of azidomethylphenylsulfide. The bottom set of images represents the detection of both signals with the application of the blocking step by means of 2 mM of azidomethylphenylsulfide. Bar: 20 µm. Adapted with permission from Liboska et al., 2012, PLoS ONE 7(12): e51679 [100].

Although EdU can be used together with BrdU for the experiments where two consecutive labeling pulses are necessary, the anti-BrdU antibody clones that do not cross-react with EdU have to be used in this case. Unfortunately, most of the anti-BrdU antibodies cross-react with EdU (Figure 7a) [100]. A rare example of an anti-BrdU antibody not reacting with EdU is the clone MoBu-1 [100,101]. Alternatively, it is possible to eliminate the unwanted reaction between EdU and anti-BrdU antibodies by using non-fluorescent azido molecules (Figure 7b) [100].

EdU was also used as an electron microscopic marker of replication. In this respect, e.g., the so-called click-EM approach was published [102]. In this approach, EdU-labeled DNA is coupled with a fluorescently-labeled azide via click reaction. Next, the photooxidation-based polymerization of diaminobenzidine (DAB) is performed, and the samples are then incubated with osmium tetroxide for visualization with an electron microscope. Although this method does not require the permeabilization step, the signal analysis requires the careful analysis of the control non-labeled cells for clear signal identification. Moreover, as the click reaction using copper ions causes partial damage of the DNA, and the above-mentioned steps are performed before the embedding step, this fact has to be taken into account as well [42,103,104,105]. Another possibility is the use e.g., of biotin-labeled azides, followed by the antibody detection step.

The application of EdU was also reported in the case of cell cycle analyses using flow cytometry [106,107,108]. Another application, where EdU can substitute BrdU, is its use for studying the replication banding pattern, which is useful, for example, to study the structure of chromosomes [109]. EdU was also used for the detection of mitochondrial DNA synthesis. Surprisingly, in contrast to BrdU, the EdU-based approaches commonly require an amplification step [71,110].

Based on EdU and click reaction, the method for the purification of proteins bound to replicated DNA was elaborated [111]. This is based on the labeling of replicated DNA by EdU and its conjugation with biotinylated azide via click reaction. After sonication, the biotin-tagged fragments of DNA are isolated using streptavidin-coated beads. This ensures that only the nascent, EdU-labeled (replicated) DNA is purified. Before the click reaction, the samples are fixed with formaldehyde, which ensures the cross-link between the DNA and the proteins. Therefore, the EdU-labeled DNA is purified together with the cross-linked proteins. These proteins are then eluted and analyzed [111]. This method allows the study of the proteins related to DNA replication.

A modified approach for DNA combing using combination of BrdU and EdU instead of IdU and CldU was also published. The improved DNA combing approach enables, according to authors, the analysis of DNA replication on single DNA molecules up to 12 Mb in length [112].

### 4.2. Other Marker Nucleosides

The toxicity of EdU led to the effort to find alternative marker nucleosides. Already in 2011, 5-ethynyl-2′-deoxycytidine (EdC) was tested for this purpose. It exhibited lower toxicity than EdU [113]. The later published data, however, showed that EdC is commonly deaminated to EdU, and the whole or at least most of the signal in DNA comes from this [114]. The lower toxicity of EdC comparing to EdU is also accompanied by a lower signal. To obtain a comparable signal, it is necessary to use higher concentrations of EdC; however, EdC toxicity increases correspondingly [114].

Neef and Luedtke presented another modified nucleoside as an alternative to EdU. They used (2′S)-2′-deoxy-2′-fluoro-5-ethynyluridine (F-ara-EdU). According to the authors, it exhibited only a minimal impact on genome function [115]. The published data also indicated that F-ara-EdU is less toxic than EdU, and is therefore more suitable for long-term experiments. Its detection was also fully compatible with BrdU labeling in pulse-chase experiments [115].

In addition to the analogs of deoxyuridine, the analogs of deoxyadenine or deoxyguanine conjugated with the ethynyl group were successfully tested for the labeling of replicating DNA [116]. Another successfully-tested, modified nucleoside was bis(POM)-phosphorylated EdU (PEdU), that was also less toxic than EdU [117].

Although all the aforementioned nucleoside analogs exhibit lower toxicity than EdU according to the authors, their use as compared to EdU or BrdU is very limited. One of the reasons can be the fact that all these analogs require copper ions for their detection, as is the case of EdU. In this respect, the higher EdU toxicity can be tolerated in principle, as most of the applications require a relatively short incubation time with EdU. Furthermore, it is not completely clear to what extent the use of these analogs is universal in terms of the effectiveness of their incorporation by various biological systems.

### 4.3. Copper Free Click Reaction

As the Cu(I)-catalyzed [3 + 2] cycloaddition exhibited the aforementioned drawbacks, alternative chemical reactions for the detection of modified nucleosides were investigated. One promising way is the inverse electron-demand Diels-Alder reaction. This type of bioorthogonal reaction uses the reaction between the electron-deficient tetrazines and electron-rich dienophiles [118]. This reaction does not require a catalyst, and can be performed in cell media [118]. Rieder and Luedtke showed that 5-vinyl-2′-deoxyurine (vinyl-dU) can be detected by this reaction [118]. Although the authors also showed that vinyl-dU is efficiently incorporated into DNA, where it can be detected by the alkene-tetrazine ligation, this method is also not widely used. This may be due to the fact that the labeled DNA has to be denaturated with 2 M HCl before alkene-tetrazine ligation [118].

## 5. Alternative Approaches of Labeling DNA Replication

Additional methods based on biotin-, digoxigenin-, or fluorochrome-tagged nucleotides are also used to label replicated DNA. In contrast to the labeled nucleosides, marker nucleotides require specific steps for their introduction into cells e.g., [119,120]. Biotin- or digoxigenin-tagged nucleotides are visualized using labeled antibodies or streptavidin. No such step is necessary if fluorochrome-tagged nucleotides are used. The advantage is that these nucleotide analogs do not require the special DNA treatment, and if a fluorochrome-tagged nucleotide is used, live cell imagining can be performed. On the other hand, the need of the specific step for their introduction into cells and the relatively high price of these nucleotides restrict their routine use.

**Figure 8:**
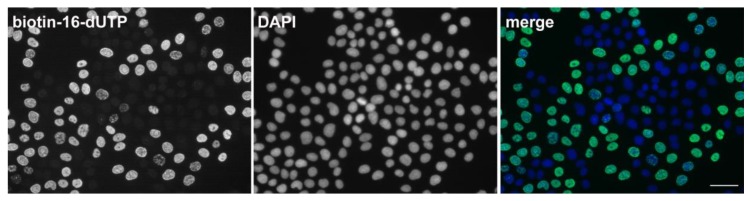
An example of labeling of DNA replication using biotin tagged deoxyuridine triphosphate. HeLa cells were hypotonically treated according to [119,121], fixed (2% formaldehyde, 10 min) and permeabilized (0.2% Triton X-100, 10 min). The biotin was detected using an anti-biotin primary antibody followed by the Alexa Fluor 488 anti-rabbit antibody (green in the color figure). The DNA was stained by DAPI (blue in the color figure). Bar: 50 µm.

The whole range of the approaches of the delivery of modified nucleotides into cells was developed. An example is a simple method of the hypotonic delivery of modified nucleotides into cells [119]. The cells are exposed to a hypotonic buffer with a marker nucleotide for several minutes, and then the cells are returned to the cultivation medium. This method allows the entry of various molecules of a molecular weight of up to several hundred Daltons without any impact on the cell viability (Figure 8) [119].

The hypotonic treatment is also an important part of the method based on the osmotic lysis of pinosomes [122]. In this case, cells are incubated in a medium with sucrose, polyethylene glycol 1000, and the modified nucleotide. Gradually, the pinocytic vesicles are formed. After the incubation of the cells in the hypotonic medium, the vesicles are broken, and their content is released [122,123]. Although these methods are relatively simple and cheap, do not require any special equipment, and allow the simultaneous affection of a high number of cells, the amount of the transported modified nucleotide is generally lower than in the case of the other approaches [123].

In this respect, the methods based on special equipment can assure much higher efficiency. A typical example is the technique of microinjection. This enables the transport of relatively large molecules into cells. This approach is based on the use of small glass capillaries and allows the transport of almost any type of macromolecules in the cell cytoplasm or nucleus [124]. The microinjection technique was used, e.g., for the visualization of replication sites in vivo [125] or for the live-cell visualization of individual chromosome territories in human cells [126]. Although microinjection enables the introduction of a high concentration of marker nucleotides, it is a relatively time-consuming approach. In addition, it requires special equipment, trained personnel, and cannot be used if a very high number of labeled cells is necessary [127]. Moreover, microinjection can lead to the cell cycle arrest or delay, or cell death in a part of the microinjected cells [123].

Another possibility of the delivery of marker nucleotides is a method based on glass beads and mechanical stress. This method also allows the transport of labeled nucleotide analogs into the cells [123,128], and enabled the analysis of individual DNA strands in living cells [128].

Concurrently, various substances facilitating the transport of marker nucleotides across the cell membrane were tested. However, not all these methods have yielded convincing results. For example, Zink and colleagues tested the delivery of modified nucleotides by transfection and lipofectamin. However, the authors obtained mainly labeling in the cytoplasm, but not in the cell nuclei [120]. On the other hand, Maya-Mendoza and colleagues successfully used the FuGene system for the delivery of modified triphosphates into cells [129,130].

In this respect, a very promising approach was developed by Zawada and colleagues [131]. They showed that the metabolically-active dNTPs (deoxyribonucleotide triphosphates) can be directly transported into cells by means of designed synthetic nucleoside triphosphate transporters. The transporters are composed of a receptor forming a non-covalent complex with a triphosphate anion, and a cell-penetrating agent translocating the complex across the plasma membrane [131].

Marker nucleotides were also used for the electron microscopic applications in various studies dealing with DNA synthesis. In this respect, biotin-16-dUTP as a marker of DNA replication was used to describe the microarchitecture of replication domains [132], replication sites in various parts of the S phase [41] or the organization of the human replicon [121].

## 6. Concluding Remarks

Considering the number of various protocols available for the detection of DNA synthesis and cellular proliferation, it is not easy to find and choose the optimal marker nucleoside and/or detection protocol for the specific purpose of given study.

The detection of nucleosides by radioisotopes using autoradiography does not usually provide sufficient resolution, is slow, and requires the observation of safety rules. In addition, the deleterious effects of H^3^-thymidine on cultured, mammalian cells were also reported [133,134,135]. Although autoradiography provided important data about the structure-functional organization of DNA replication, its recent use is very limited.

In this respect, the protocols based on EdU or BrdU represent the optimal choice for most of the applications such as light microscopy analyses or flow cytometry applications. Both nucleosides are effectively incorporated into the nuclear DNA of replicating cells, there is no need for specific equipment, both are commercially available, they are relatively cheap, and the protocols for their detection are quick and have been described in great detail.

Some published data indicates that BrdU is more sensitive than EdU. In this respect, the short pulses (5 min) with the marker nucleoside result in the better resolution of replicated cells if BrdU is used [47,48]. This finding is in agreement with the higher sensitivity of BrdU-based approach in the case of the detection of DNA synthesis in mitochondria [42] and results of DNA combing, when fainter signal is observed if EdU is used [136]. Concerning the use of BrdU and EdU on the tissue sections, EdU is probably a better choice than BrdU. Due to the small size of fluorescently-labeled azides, relatively thick tissue sections can be used, and the whole mount specimens can be labeled more readily and reliably than in the case of BrdU [137,138]. Diermeier-Daucher and colleagues compared EdU and BrdU usage in the flow cytometry in terms of cell cycle kinetics, cell viability, and DNA damage. They found only minor differences between these two nucleosides, although the cell viability was affected by the long incubation of cells with EdU [108].

If long labeling pulses or long pulse-chase experiments are necessary, BrdU should be used, as it has usually exhibited much lower toxicity than EdU [115]. BrdU is also more suitable in experiments when the concurrent detection of fluorescent proteins such as GFP is required [47]. EdU is also unsuitable in experiments where the cells are synchronized using elevated concentrations of thymidine, or if the pulse with the marker nucleoside should be performed immediately after the BrdU pulse, as it results in the nearly complete omission of the EdU signal or its significant decrease. The high intracellular concentrations of thymidine or BrdU probably compete with EdU, and stop or reduce its incorporation into DNA for some time. In this respect, in double labeling experiments with BrdU and EdU, the EdU pulse should precede the BrdU pulse. It especially concerns cases if there is no or only a very short chase period between pulses, or if thymidine is used during the chase to stop the incorporation of the first analogue.

Not one of these protocols can be used for live cell imaging, as BrdU requires cell permeabilization and DNA treatment and EdU is visualized by a click reaction using highly toxic copper ions. Although vinyl-dU can be used instead of EdU and a copper-free click reaction can be used for its detection, the DNA treatment by acid is necessary before this step [118]. In this respect, if this approach could be optimized in terms of the omission of HCl, it has the potential to compete with EdU and BrdU detection.

The techniques based on nucleotides conjugated with various fluorochromes are the only choice for live cell imaging of replicated DNA. However, these nucleotides require protocols for their effective delivery into cells. In this regard, the protocol based on the use of nucleotide transporters may be the optimal choice, as it exhibits high efficiency and low toxicity [131]. On the other hand, one has to keep in mind that it is crucial for cells to have dNTPs levels that are optimal for DNA replication [139]. In this respect, the direct introduction of high or low concentrations of even natural nucleotides can lead to the imbalance in the nucleotide pools, resulting in, for example, the acceleration of the replication fork [54], the propensity of DNA polymerases to extend a mismatched primer-template, reduced efficiency of proofreading at high nucleotide levels [139,140,141], and in a decrease of the length of S phase or inhibition of DNA replication and fork stalling [139]. Therefore, proper control should accompany the particular experiments.

Although it is very difficult to estimate the future development of the marker nucleosides and nucleotides, it seems that the current progresses in copper free click chemistry [118] and specific nucleotide transporters [131] can yield new methods for the detection of DNA replication and cellular proliferation in both living and fixed cells.
